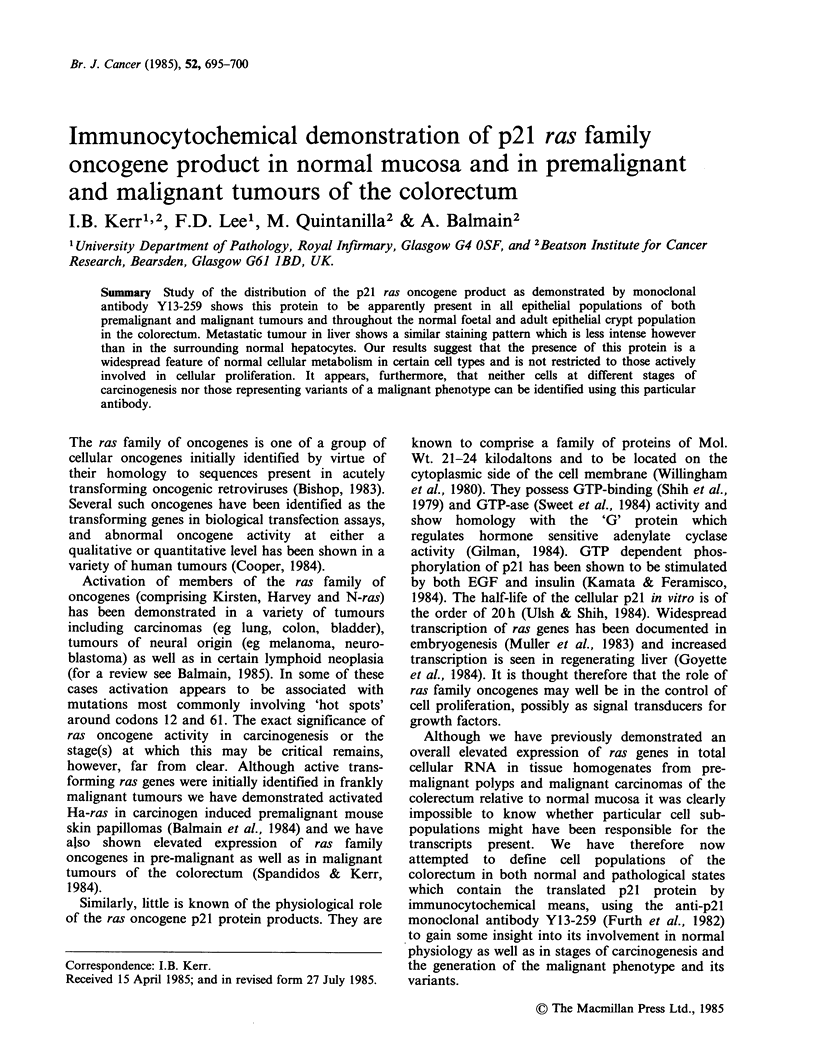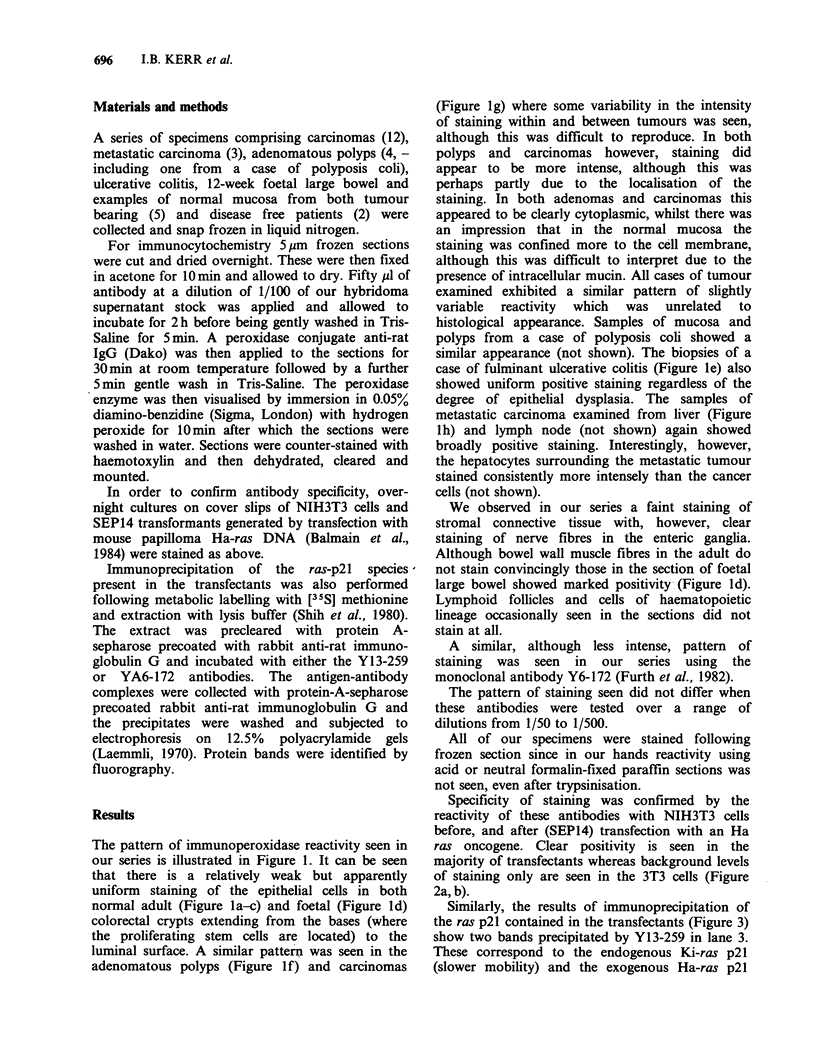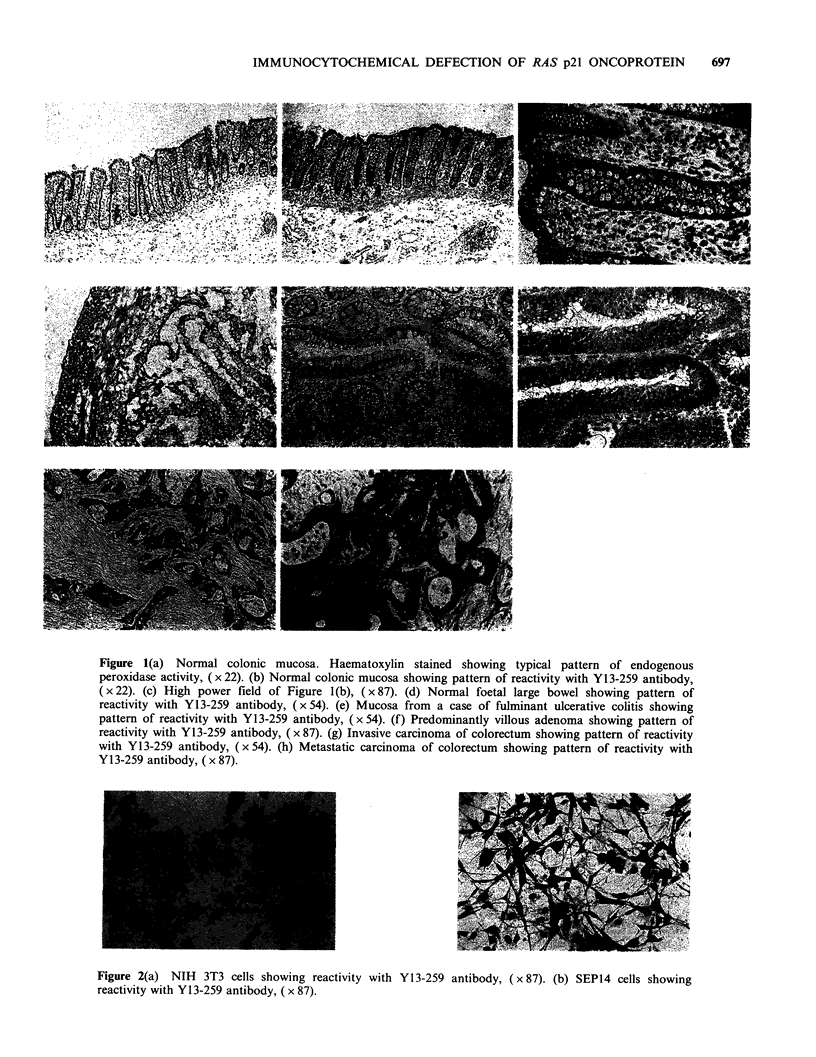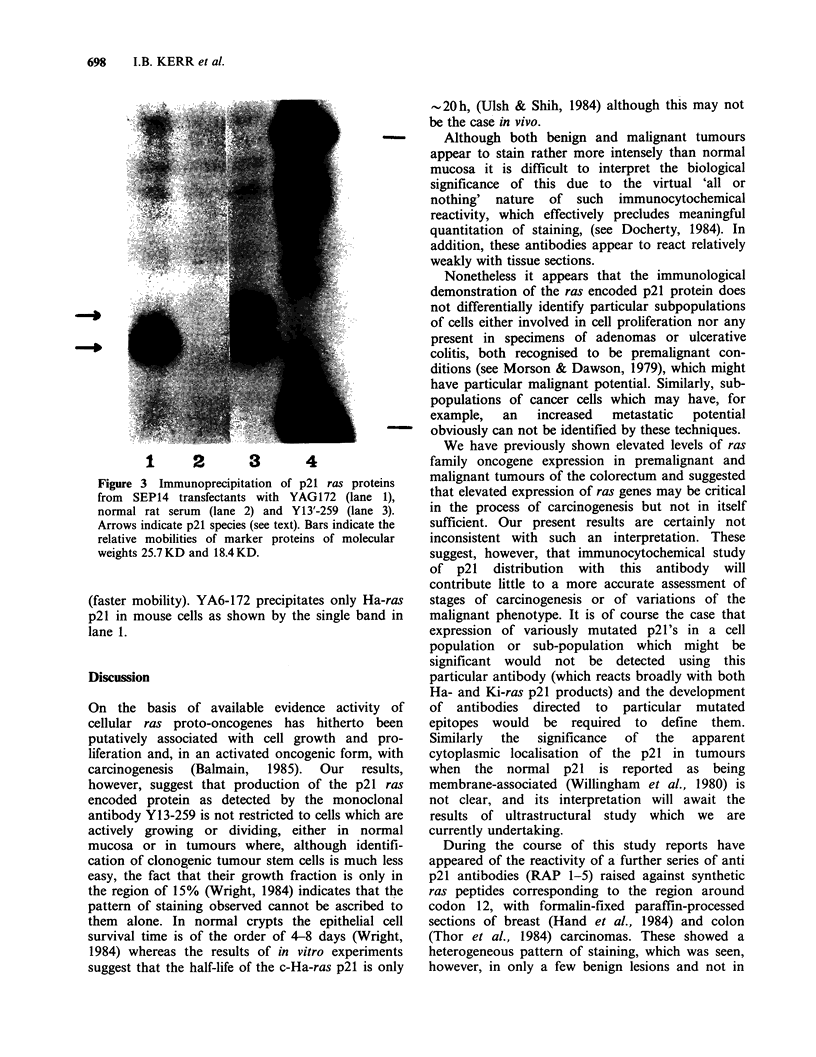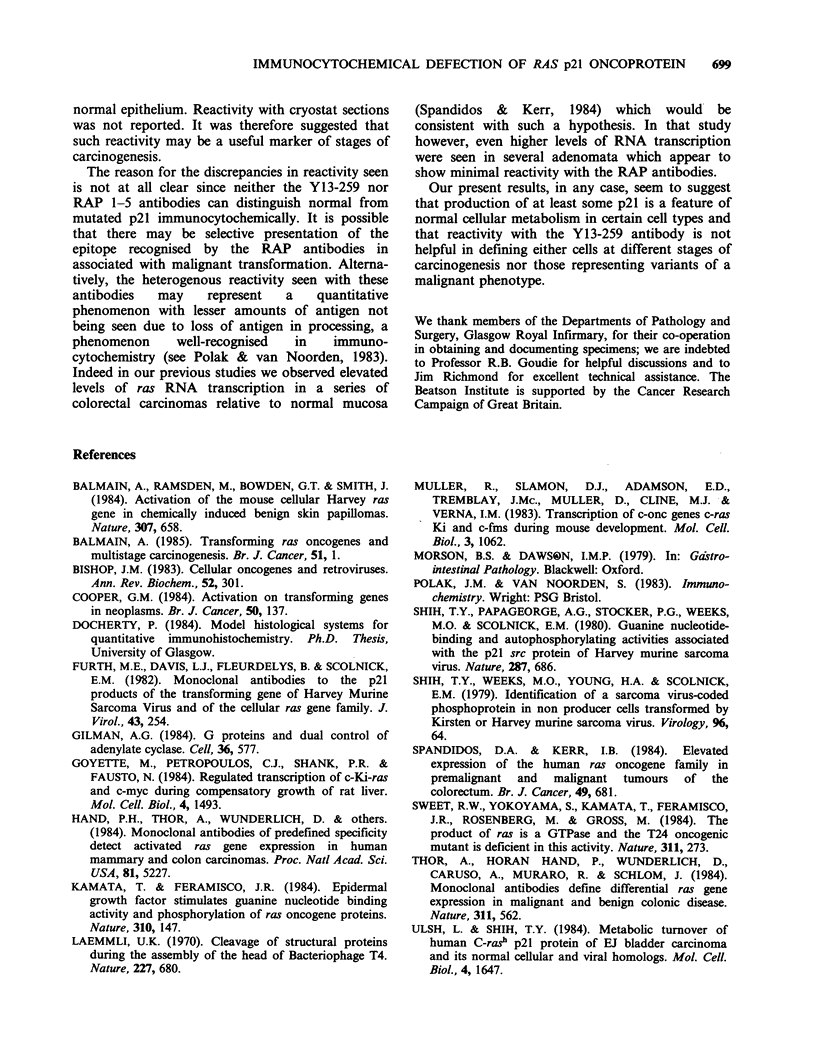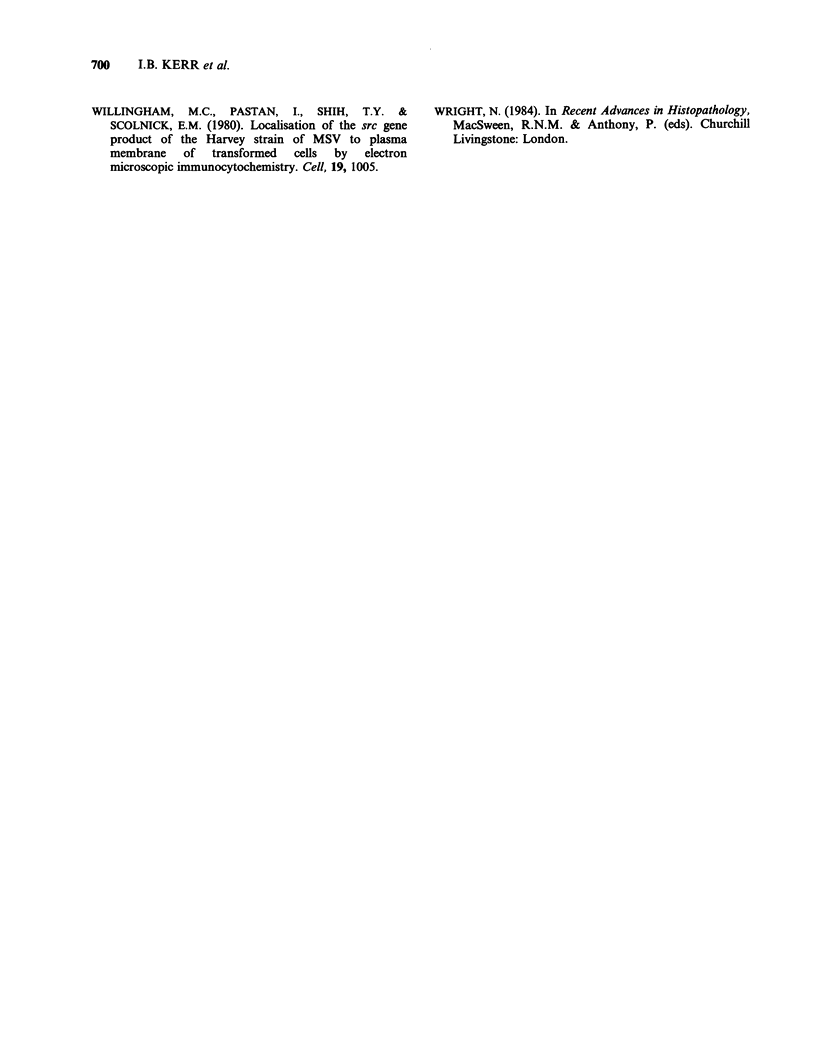# Immunocytochemical demonstration of p21 ras family oncogene product in normal mucosa and in premalignant and malignant tumours of the colorectum.

**DOI:** 10.1038/bjc.1985.245

**Published:** 1985-11

**Authors:** I. B. Kerr, F. D. Lee, M. Quintanilla, A. Balmain

## Abstract

**Images:**


*Br. J. Cancer* (1985), **52**, 695–700

### Immunocytochemical demonstration of p21 *ras* family oncogene product in normal mucosa and in premalignant and malignant tumours of the colorectum

I.B. Kerr[1,2], F.D. Lee[1], M. Quintanilla[2] & A. Balmain[2]

[1]*University Department of Pathology, Royal Infirmary, Glasgow G4 0SF, and* [2]*Beatson Institute for Cancer Research, Bearsden, Glasgow G61 1BD, UK.*

**Summary** Study of the distribution of the p21 *ras* oncogene product as demonstrated by monoclonal antibody Y13-259 shows this protein to be apparently present in all epithelial populations of both premalignant and malignant tumours and throughout the normal foetal and adult epithelial crypt population in the colorectum. Metastatic tumour in liver shows a similar staining pattern which is less intense however than in the surrounding normal hepatocytes. Our results suggest that the presence of this protein is a widespread feature of normal cellular metabolism in certain cell types and is not restricted to those actively involved in cellular proliferation. It appears, furthermore, that neither cells at different stages of carcinogenesis nor those representing variants of a malignant phenotype can be identified using this particular antibody.

The *ras* family of oncogenes is one of a group of cellular oncogenes initially identified by virtue of their homology to sequences present in acutely transforming oncogenic retroviruses (Bishop, 1983). Several such oncogenes have been identified as the transforming genes in biological transfection assays, and abnormal oncogene activity at either a qualitative or quantitative level has been shown in a variety of human tumours (Cooper, 1984).

Activation of members of the *ras* family of oncogenes (comprising Kirsten, Harvey and N-*ras*) has been demonstrated in a variety of tumours including carcinomas (eg lung, colon, bladder), tumours of neural origin (eg melanoma, neuroblastoma) as well as in certain lymphoid neoplasia (for a review see Balmain, 1985). In some of these cases activation appears to be associated with mutations most commonly involving 'hot spots' around codons 12 and 61. The exact significance of *ras* oncogene activity in carcinogenesis or the stage(s) at which this may be critical remains, however, far from clear. Although active transforming *ras* genes were initially identified in frankly malignant tumours we have demonstrated activated Ha-*ras* in carcinogen induced premalignant mouse skin papillomas (Balmain *et al.*, 1984) and we have also shown elevated expression of *ras* family oncogenes in pre-malignant as well as in malignant tumours of the colorectum (Spandidos & Kerr, 1984).

Similarly, little is known of the physiological role of the *ras* oncogene p21 protein products. They are known to comprise a family of proteins of Mol. Wt. 21–24 kilodaltons and to be located on the cytoplasmic side of the cell membrane (Willingham *et al.*, 1980). They possess GTP-binding (Shih *et al.*, 1979) and GTP-ase (Sweet *et al.*, 1984) activity and show homology with the 'G' protein which regulates hormone sensitive adenylate cyclase activity (Gilman, 1984). GTP dependent phosphorylation of p21 has been shown to be stimulated by both EGF and insulin (Kamata & Feramisco, 1984). The half-life of the cellular p21 *in vitro* is of the order of 20 h (Ulsh & Shih, 1984). Widespread transcription of *ras* genes has been documented in embryogenesis (Muller *et al.*, 1983) and increased transcription is seen in regenerating liver (Goyette *et al.*, 1984). It is thought therefore that the role of *ras* family oncogenes may well be in the control of cell proliferation, possibly as signal transducers for growth factors.

Although we have previously demonstrated an overall elevated expression of *ras* genes in total cellular RNA in tissue homogenates from premalignant polyps and malignant carcinomas of the colerectum relative to normal mucosa it was clearly impossible to know whether particular cell subpopulations might have been responsible for the transcripts present. We have therefore now attempted to define cell populations of the colorectum in both normal and pathological states which contain the translated p21 protein by immunocytochemical means, using the anti-p21 monoclonal antibody Y13-259 (Furth *et al.*, 1982) to gain some insight into its involvement in normal physiology as well as in stages of carcinogenesis and the generation of the malignant phenotype and its variants.

Correspondence: I.B. Kerr.
Received 15 April 1985; and in revised form 27 July 1985.

© The Macmillan Press Ltd., 1985

### Materials and methods

A series of specimens comprising carcinomas (12), metastatic carcinoma (3), adenomatous polyps (4, – including one from a case of polyposis coli), ulcerative colitis, 12-week foetal large bowel and examples of normal mucosa from both tumour bearing (5) and disease free patients (2) were collected and snap frozen in liquid nitrogen.

For immunocytochemistry 5 $\mu$m frozen sections were cut and dried overnight. These were then fixed in acetone for 10 min and allowed to dry. Fifty $\mu$l of antibody at a dilution of 1/100 of our hybridoma supernatant stock was applied and allowed to incubate for 2 h before being gently washed in Tris-Saline for 5 min. A peroxidase conjugate anti-rat IgG (Dako) was then applied to the sections for 30 min at room temperature followed by a further 5 min gentle wash in Tris-Saline. The peroxidase enzyme was then visualised by immersion in 0.05% diamino-benzidine (Sigma, London) with hydrogen peroxide for 10 min after which the sections were washed in water. Sections were counter-stained with haemotoxylin and then dehydrated, cleared and mounted.

In order to confirm antibody specificity, overnight cultures on cover slips of NIH3T3 cells and SEP14 transformants generated by transfection with mouse papilloma Ha-*ras* DNA (Balmain *et al.*, 1984) were stained as above.

Immunoprecipitation of the *ras*-p21 species present in the transfectants was also performed following metabolic labelling with [$^{35}$S] methionine and extraction with lysis buffer (Shih *et al.*, 1980). The extract was precleared with protein A-sepharose precoated with rabbit anti-rat immunoglobulin G and incubated with either the Y13-259 or YA6-172 antibodies. The antigen-antibody complexes were collected with protein-A-sepharose precoated rabbit anti-rat immunoglobulin G and the precipitates were washed and subjected to electrophoresis on 12.5% polyacrylamide gels (Laemmli, 1970). Protein bands were identified by fluorography.

### Results

The pattern of immunoperoxidase reactivity seen in our series is illustrated in Figure 1. It can be seen that there is a relatively weak but apparently uniform staining of the epithelial cells in both normal adult (Figure 1a–c) and foetal (Figure 1d) colorectal crypts extending from the bases (where the proliferating stem cells are located) to the luminal surface. A similar pattern was seen in the adenomatous polyps (Figure 1f) and carcinomas (Figure 1g) where some variability in the intensity of staining within and between tumours was seen, although this was difficult to reproduce. In both polyps and carcinomas however, staining did appear to be more intense, although this was perhaps partly due to the localisation of the staining. In both adenomas and carcinomas this appeared to be clearly cytoplasmic, whilst there was an impression that in the normal mucosa the staining was confined more to the cell membrane, although this was difficult to interpret due to the presence of intracellular mucin. All cases of tumour examined exhibited a similar pattern of slightly variable reactivity which was unrelated to histological appearance. Samples of mucosa and polyps from a case of polyposis coli showed a similar appearance (not shown). The biopsies of a case of fulminant ulcerative colitis (Figure 1e) also showed uniform positive staining regardless of the degree of epithelial dysplasia. The samples of metastatic carcinoma examined from liver (Figure 1h) and lymph node (not shown) again showed broadly positive staining. Interestingly, however, the hepatocytes surrounding the metastatic tumour stained consistently more intensely than the cancer cells (not shown).

We observed in our series a faint staining of stromal connective tissue with, however, clear staining of nerve fibres in the enteric ganglia. Although bowel wall muscle fibres in the adult do not stain convincingly those in the section of foetal large bowel showed marked positivity (Figure 1d). Lymphoid follicles and cells of haematopoietic lineage occasionally seen in the sections did not stain at all.

A similar, although less intense, pattern of staining was seen in our series using the monoclonal antibody Y6-172 (Furth *et al.*, 1982).

The pattern of staining seen did not differ when these antibodies were tested over a range of dilutions from 1/50 to 1/500.

All of our specimens were stained following frozen section since in our hands reactivity using acid or neutral formalin-fixed paraffin sections was not seen, even after trypsinisation.

Specificity of staining was confirmed by the reactivity of these antibodies with NIH3T3 cells before, and after (SEP14) transfection with an Ha *ras* oncogene. Clear positivity is seen in the majority of transfectants whereas background levels of staining only are seen in the 3T3 cells (Figure 2a, b).

Similarly, the results of immunoprecipitation of the *ras* p21 contained in the transfectants (Figure 3) show two bands precipitated by Y13-259 in lane 3. These correspond to the endogenous Ki-*ras* p21 (slower mobility) and the exogenous Ha-*ras* p21

**Figure** 1(a) Normal colonic mucosa. Haematoxylin stained showing typical pattern of endogenous peroxidase activity, (×22). (b) Normal colonic mucosa showing pattern of reactivity with Y13-259 antibody, (×22). (c) High power field of Figure 1(b), (×87). (d) Normal foetal large bowel showing pattern of reactivity with Y13-259 antibody, (×54). (e) Mucosa from a case of fulminant ulcerative colitis showing pattern of reactivity with Y13-259 antibody, (×54). (f) Predominantly villous adenoma showing pattern of reactivity with Y13-259 antibody, (×87). (g) Invasive carcinoma of colorectum showing pattern of reactivity with Y13-259 antibody, (×54). (h) Metastatic carcinoma of colorectum showing pattern of reactivity with Y13-259 antibody, (×87).

**Figure** 2(a) NIH 3T3 cells showing reactivity with Y13-259 antibody, (×87). (b) SEP14 cells showing reactivity with Y13-259 antibody, (×87).

**Figure 3** Immunoprecipitation of p21 *ras* proteins from SEP14 transfectants with YAG172 (lane 1), normal rat serum (lane 2) and Y13′-259 (lane 3). Arrows indicate p21 species (see text). Bars indicate the relative mobilities of marker proteins of molecular weights 25.7 KD and 18.4 KD.

(faster mobility). YA6-172 precipitates only Ha-*ras* p21 in mouse cells as shown by the single band in lane 1.

### Discussion

On the basis of available evidence activity of cellular *ras* proto-oncogenes has hitherto been putatively associated with cell growth and proliferation and, in an activated oncogenic form, with carcinogenesis (Balmain, 1985). Our results, however, suggest that production of the p21 *ras* encoded protein as detected by the monoclonal antibody Y13-259 is not restricted to cells which are actively growing or dividing, either in normal mucosa or in tumours where, although identification of clonogenic tumour stem cells is much less easy, the fact that their growth fraction is only in the region of 15% (Wright, 1984) indicates that the pattern of staining observed cannot be ascribed to them alone. In normal crypts the epithelial cell survival time is of the order of 4–8 days (Wright, 1984) whereas the results of *in vitro* experiments suggest that the half-life of the c-Ha-*ras* p21 is only ~20 h, (Ulsh & Shih, 1984) although this may not be the case *in vivo*.

Although both benign and malignant tumours appear to stain rather more intensely than normal mucosa it is difficult to interpret the biological significance of this due to the virtual 'all or nothing' nature of such immunocytochemical reactivity, which effectively precludes meaningful quantitation of staining, (see Docherty, 1984). In addition, these antibodies appear to react relatively weakly with tissue sections.

Nonetheless it appears that the immunological demonstration of the *ras* encoded p21 protein does not differentially identify particular subpopulations of cells either involved in cell proliferation nor any present in specimens of adenomas or ulcerative colitis, both recognised to be premalignant conditions (see Morson & Dawson, 1979), which might have particular malignant potential. Similarly, subpopulations of cancer cells which may have, for example, an increased metastatic potential obviously can not be identified by these techniques.

We have previously shown elevated levels of *ras* family oncogene expression in premalignant and malignant tumours of the colorectum and suggested that elevated expression of *ras* genes may be critical in the process of carcinogenesis but not in itself sufficient. Our present results are certainly not inconsistent with such an interpretation. These suggest, however, that immunocytochemical study of p21 distribution with this antibody will contribute little to a more accurate assessment of stages of carcinogenesis or of variations of the malignant phenotype. It is of course the case that expression of variously mutated p21's in a cell population or sub-population which might be significant would not be detected using this particular antibody (which reacts broadly with both Ha- and Ki-*ras* p21 products) and the development of antibodies directed to particular mutated epitopes would be required to define them. Similarly the significance of the apparent cytoplasmic localisation of the p21 in tumours when the normal p21 is reported as being membrane-associated (Willingham *et al.*, 1980) is not clear, and its interpretation will await the results of ultrastructural study which we are currently undertaking.

During the course of this study reports have appeared of the reactivity of a further series of anti p21 antibodies (RAP 1–5) raised against synthetic *ras* peptides corresponding to the region around codon 12, with formalin-fixed paraffin-processed sections of breast (Hand *et al.*, 1984) and colon (Thor *et al.*, 1984) carcinomas. These showed a heterogeneous pattern of staining, which was seen, however, in only a few benign lesions and not in normal epithelium. Reactivity with cryostat sections was not reported. It was therefore suggested that such reactivity may be a useful marker of stages of carcinogenesis.

The reason for the discrepancies in reactivity seen is not at all clear since neither the Y13-259 nor RAP 1–5 antibodies can distinguish normal from mutated p21 immunocytochemically. It is possible that there may be selective presentation of the epitope recognised by the RAP antibodies in associated with malignant transformation. Alternatively, the heterogenous reactivity seen with these antibodies may represent a quantitative phenomenon with lesser amounts of antigen not being seen due to loss of antigen in processing, a phenomenon well-recognised in immunocytochemistry (see Polak & van Noorden, 1983). Indeed in our previous studies we observed elevated levels of *ras* RNA transcription in a series of colorectal carcinomas relative to normal mucosa (Spandidos & Kerr, 1984) which would be consistent with such a hypothesis. In that study however, even higher levels of RNA transcription were seen in several adenomata which appear to show minimal reactivity with the RAP antibodies.

Our present results, in any case, seem to suggest that production of at least some p21 is a feature of normal cellular metabolism in certain cell types and that reactivity with the Y13-259 antibody is not helpful in defining either cells at different stages of carcinogenesis nor those representing variants of a malignant phenotype.

We thank members of the Departments of Pathology and Surgery, Glasgow Royal Infirmary, for their co-operation in obtaining and documenting specimens; we are indebted to Professor R.B. Goudie for helpful discussions and to Jim Richmond for excellent technical assistance. The Beatson Institute is supported by the Cancer Research Campaign of Great Britain.

### References

BALMAIN, A., RAMSDEN, M., BOWDEN, G.T. & SMITH, J. (1984). Activation of the mouse cellular Harvey *ras* gene in chemically induced benign skin papillomas. *Nature*, **307**, 658.

BALMAIN, A. (1985). Transforming *ras* oncogenes and multistage carcinogenesis. *Br. J. Cancer*, **51**, 1.

BISHOP, J.M. (1983). Cellular oncogenes and retroviruses. *Ann. Rev. Biochem.*, **52**, 301.

COOPER, G.M. (1984). Activation on transforming genes in neoplasms. *Br. J. Cancer*, **50**, 137.

DOCHERTY, P. (1984). Model histological systems for quantitative immunohistochemistry. *Ph.D. Thesis*, University of Glasgow.

FURTH, M.E., DAVIS, L.J., FLEURDELYS, B. & SCOLNICK, E.M. (1982). Monoclonal antibodies to the p21 products of the transforming gene of Harvey Murine Sarcoma Virus and of the cellular *ras* gene family. *J. Virol.*, **43**, 254.

GILMAN, A.G. (1984). G proteins and dual control of adenylate cyclase. *Cell*, **36**, 577.

GOYETTE, M., PETROPOULOS, C.J., SHANK, P.R. & FAUSTO, N. (1984). Regulated transcription of c-Ki-*ras* and c-myc during compensatory growth of rat liver. *Mol. Cell. Biol.*, **4**, 1493.

HAND, P.H., THOR, A., WUNDERLICH, D. & others. (1984). Monoclonal antibodies of predefined specificity detect activated *ras* gene expression in human mammary and colon carcinomas. *Proc. Natl Acad. Sci. USA*, **81**, 5227.

KAMATA, T. & FERAMISCO, J.R. (1984). Epidermal growth factor stimulates guanine nucleotide binding activity and phosphorylation of *ras* oncogene proteins. *Nature*, **310**, 147.

LAEMMLI, U.K. (1970). Cleavage of structural proteins during the assembly of the head of Bacteriophage T4. *Nature*, **227**, 680.

MULLER, R., SLAMON, D.J., ADAMSON, E.D., TREMBLAY, J.Mc., MULLER, D., CLINE, M.J. & VERNA, I.M. (1983). Transcription of c-onc genes c-*ras* Ki and c-fms during mouse development. *Mol. Cell. Biol.*, **3**, 1062.

MORSON, B.S. & DAWSON, I.M.P. (1979). In: *Gastro-intestinal Pathology*. Blackwell: Oxford.

POLAK, J.M. & VAN NOORDEN, S. (1983). *Immunochemistry*. Wright: PSG Bristol.

SHIH, T.Y., PAPAGEORGE, A.G., STOCKER, P.G., WEEKS, M.O. & SCOLNICK, E.M. (1980). Guanine nucleotide-binding and autophosphorylating activities associated with the p21 *src* protein of Harvey murine sarcoma virus. *Nature*, **287**, 686.

SHIH, T.Y., WEEKS, M.O., YOUNG, H.A. & SCOLNICK, E.M. (1979). Identification of a sarcoma virus-coded phosphoprotein in non producer cells transformed by Kirsten or Harvey murine sarcoma virus. *Virology*, **96**, 64.

SPANDIDOS, D.A. & KERR, I.B. (1984). Elevated expression of the human *ras* oncogene family in premalignant and malignant tumours of the colorectum. *Br. J. Cancer*, **49**, 681.

SWEET, R.W., YOKOYAMA, S., KAMATA, T., FERAMISCO, J.R., ROSENBERG, M. & GROSS, M. (1984). The product of *ras* is a GTPase and the T24 oncogenic mutant is deficient in this activity. *Nature*, **311**, 273.

THOR, A., HORAN HAND, P., WUNDERLICH, D., CARUSO, A., MURARO, R. & SCHLOM, J. (1984). Monoclonal antibodies define differential *ras* gene expression in malignant and benign colonic disease. *Nature*, **311**, 562.

ULSH, L. & SHIH, T.Y. (1984). Metabolic turnover of human C-*ras*$^{h}$ p21 protein of EJ bladder carcinoma and its normal cellular and viral homologs. *Mol. Cell. Biol.*, **4**, 1647.

WILLINGHAM, M.C., PASTAN, I., SHIH, T.Y. & SCOLNICK, E.M. (1980). Localisation of the *src* gene product of the Harvey strain of MSV to plasma membrane of transformed cells by electron microscopic immunocytochemistry. *Cell*, **19**, 1005.

WRIGHT, N. (1984). In *Recent Advances in Histopathology*, MacSween, R.N.M. & Anthony, P. (eds). Churchill Livingstone: London.